# Deep learning-driven automated detection of canine cardiac murmurs via digital wireless stethoscope auscultation

**DOI:** 10.1007/s11259-026-11131-5

**Published:** 2026-03-03

**Authors:** Sully Lee, HyeSun Chang, Won-Yang Cho, Soyeon Jeon, Sangjun Lee, Sehoon Kim, Min-Ok Ryu, Kyoung-Won Seo

**Affiliations:** 1 Laboratory of Veterinary Internal Medicine, Department of Veterinary Clinical Science, College of Veterinary Medicine, Seoul National University, Seoul, Republic of Korea; 2 Smartsound Corporation, Seoul, Republic of Korea; 3 Department of AI-SW Convergence, Soongsil University, Seoul, Republic of Korea

**Keywords:** Deep learning, Murmur, Canine, Myxomatous mitral valve disease, Digital stethoscope, CNN6

## Abstract

A heart murmur is a key indicator of cardiovascular disease, making auscultation essential. Nonetheless, its accuracy is influenced by the clinician’s experience and subjective interpretation. This study developed a deep learning–based algorithm (CNN6) for automated detection of heart murmurs using phonocardiogram (PCG) data acquired via a digital wireless stethoscope. A total of 2,269 recordings (over 20 h) from 406 dogs were used for model development and validation, and 297 recordings from 60 dogs were reserved for independent testing. The model achieved 89.9% sensitivity, 92.7% specificity, and 90.9% accuracy, demonstrating diagnostic performance comparable to that of experienced veterinarians. This AI-assisted approach provides a consistent and objective murmur assessment and represents a clinically applicable screening tool for myxomatous mitral valve disease (MMVD), enhancing diagnostic precision, facilitating telemedicine, and promoting the integration of artificial intelligence into veterinary cardiology.

## Introduction

Cardiovascular disease is a significant health concern in dogs, particularly older animals, with myxomatous mitral valve disease (MMVD) being the most prevalent. MMVD accounts for 75–80% of all cardiac cases in dogs (Ljungvall and Häggström 2017). MMVD is a degenerative and progressive disorder that is often clinically silent in its early stages but progressively worsens over time, ultimately leading to congestive heart failure. Therefore, early detection and timely intervention are critical to improve both the prognosis and quality of life of affected dogs.

The presence of heart murmur is a principal indicator of the likelihood of cardiovascular disease, thereby making auscultation a crucial component of physical examinations (Keene et al. 2019). The severity of the murmur is indicative of progression of the underlying disease. One study (Ljungvall et al. 2014) identified murmur intensity as a reliable predictor of MMVD progression. Similarly, this correlation is observed in other major congenital heart conditions, such as pulmonary valve stenosis and subaortic stenosis, where the intensity of the murmur increases with disease severity, thereby underscoring its role as an important marker of disease progression (Caivano et al. 2018).

The assessment of heart murmurs through auscultation traditionally relies on the use of a stethoscope by veterinarians. However, this method is subject to variability because its accuracy and consistency are heavily influenced by the clinicians’ experience, skill, and subjective interpretation. Such limitations increase the risk of misdiagnosis or overlooking the early signs of heart disease, potentially leading to delays in appropriate treatment (Mullowney et al. 2021; Van Staveren and Szatmári 2020). These challenges underscore the need for more objective and quantitative diagnostic tools to enhance the precision and reliability of cardiac evaluation.

In recent years, there have been notable advancements in the fields of AI and deep learning technologies, which have opened new avenues for the automated analysis of heart sounds. Deep-learning algorithms trained on large datasets of auscultation recordings have demonstrated the ability to detect and classify heart murmurs with an accuracy comparable to that of human experts (Chorba et al. 2021; DeGroff et al. 2001; Elola et al. 2023). These technologies have the potential to standardize cardiac assessments, reduce diagnostic variability, and assist veterinarians in making more consistent and accurate diagnoses. In particular, deep learning has the potential to eliminate much of the subjectivity associated with traditional auscultation, thereby enabling less-experienced practitioners to make more confident clinical decisions, especially in rural or low-resource settings where access to specialists may be limited.

The advent of digital wireless stethoscopes has enhanced the practicality of modern auscultation techniques (Arjoune et al. 2023). These devices facilitate continuous monitoring and convenient archiving of recordings, thereby enabling the long-term tracking of disease progression. Furthermore, their seamless integration with diagnostic systems and capacity to facilitate remote collaboration with specialists markedly enhance diagnostic accuracy. These capabilities make wireless stethoscopes an optimal instrument for telemedicine, enabling veterinarians to assess cardiac status irrespective of the patient’s location (Fan et al. 2022).

Recent work demonstrated the feasibility of adapting a deep learning algorithm originally trained on human PCG data to grade heart murmurs in dogs through fine-tuning (McDonald et al. 2024). While their study highlighted the potential of cross-species transfer learning, our approach differs in that the model was trained exclusively on a large dataset of canine heart sound recordings. This species-specific training is expected to better capture the unique acoustic characteristics of canine cardiology and improve diagnostic performance in veterinary settings.

This study seeks to develop an advanced system that leverages deep learning algorithms to analyze canine cardiac auscultation data obtained through digital wireless stethoscopes, with the goal of automating the detection of heart murmurs. Such a system would provide a more objective and reliable tool in early diagnosis of heart disease in dogs, thereby supporting veterinarians and pet owners in making informed clinical decisions. By automatically analyzing heart murmur data, this system aims to overcome the inherent limitations of traditional subjective diagnostics. It is noteworthy that this research represents an inaugural study on deep learning-based canine murmur auscultation, signifying a substantial advancement in the implementation of AI technology in veterinary medicine, and offering an innovative instrument for enhancing animal healthcare management.

## Materials and methods

### Clinical study design

**Fig. 1:**
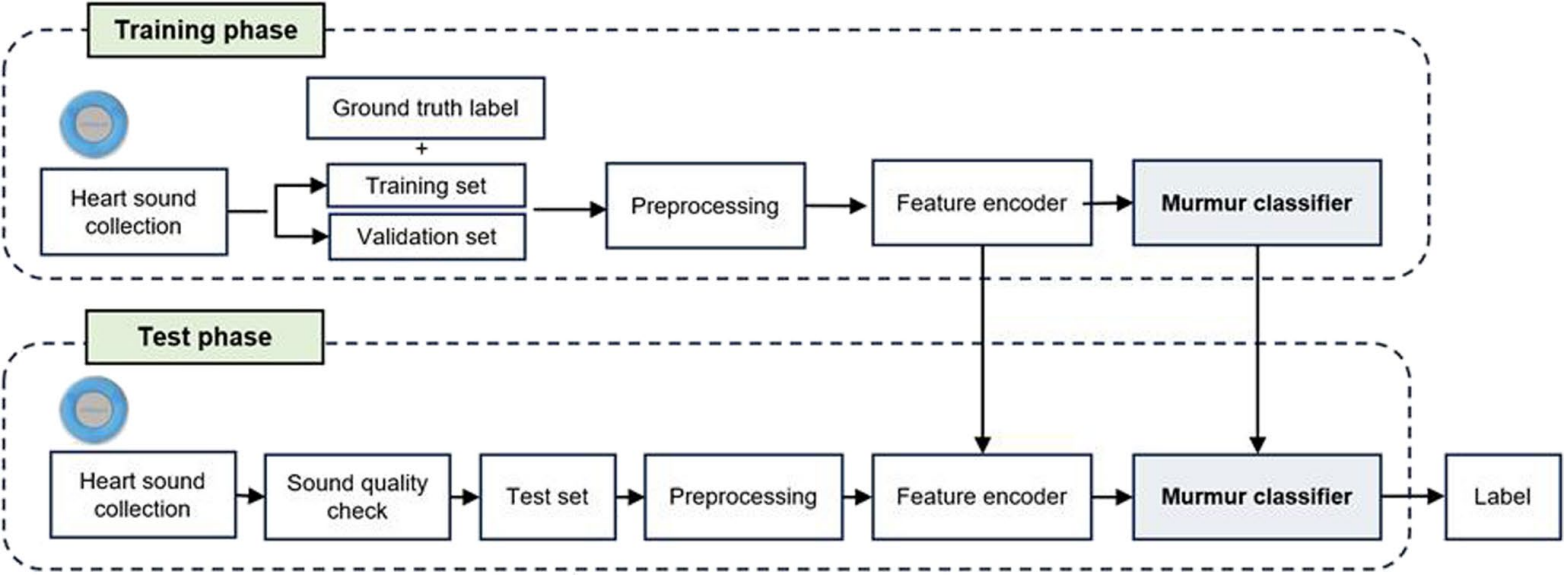
Schematic overview of the development and testing process for a deep learning model to classify heart murmurs in dogs using phonocardiogram data. This figure illustrates a systemic overview from heart sound collection, through preprocessing and feature encoding, to the development of the murmur classifier using a dataset divided into training, validation, and test sets in a 7:2:1 ratio.The model was trained on the training set, tuned with the validation set, and evaluated on the independent test set to ensure robust performance

This study aimed to develop an automated classifier for the detection of heart murmurs using PCG data (Fig. 1). Data were collected at Seoul National University Veterinary Medical Teaching Hospital from May 2022 to September 2024. This study included dogs with normal heart sounds and murmurs. Trained veterinarians classified the heart sounds using a standardized auscultation process. The owners were informed about the purpose of the study, and consent for the use of their dogs’ data was obtained. This study was approved by the Institutional Animal Care and Use Committee.

Heart sounds were sampled from enrolled dogs using a predefined protocol. The resulting PCG data served as the primary dataset, which was initially divided into training and test sets in a conventional 7:3 ratio to ensure robust model evaluation. The test set was further split into validation and test subsets in a 2:1 ratio, yielding a final data split allocation of 1972 recordings (70.0%) for training, 548 (19.5%) for validation, and 297 (10.5%) for testing. This systematic division allowed for the effective development and refinement of the deep learning model while ensuring a reliable assessment of AI performance through comparison with veterinarian evaluations.

Each dataset was composed of nonoverlapping individuals. To train the deep learning model, heart sounds collected from all auscultation sites were utilized to ensure comprehensive data coverage. As MMVD is the most prevalent form of cardiac disease in dogs and is identified through the detection of mitral valve regurgitation, the heart sounds included in the test subset were exclusively sampled from the mitral valve. This approach was designed to maximize the clinical relevance and future applicability of the proposed algorithm.

### Comprehensive data collection

#### Stethoscope recordings

Trained veterinarians conducted auscultations following a standardized protocol using either a standard or digital wireless stethoscope. The dogs were kept as calm as possible in a quiet environment to minimize rapid breathing or trembling. The auscultation sites were carefully organized, as shown in Table 1, and the stethoscope was precisely positioned at each site to ensure accurate assessment. For the digital stethoscope, the device was gently applied with minimal pressure – just enough to maintain skin contact without compressing the thoracic wall – to the auscultation sites to reduce friction noise from the dog’s movement, with heart sounds repeatedly recorded for 15–30 s per site. Any recordings with compromised sound quality owing to external interference were discarded and re-recorded. Data were included in the study even if all auscultation sites were not assessed in any dog. Recorded PCG data from the enrolled dogs were saved as 16-bit, 4000-, and 500-Hz samples in the WAV file format.

**Table 1:** Auscultation sites

Valve	Side	Position
Miral	Left	5th intercostal space Costochondral junction on the left apex
Aortic	Left	4th intercostal space Just above the costochondral junction
Pulmonic	Left	2nd-4th intercostal space Just above the sternum
Tricuspid	Right	3rd-5th intercostal space Near the costochondral junction

#### Echocardiographic data

A comprehensive echocardiographic evaluation was performed on dogs with heart murmurs detected during routine auscultation at Seoul National University Veterinary Medical Teaching Hospital. The procedure was performed by a trained veterinarian from the Department of Radiology, utilizing phased-array transducers in conjunction with a simultaneous single-lead electrocardiogram and included 2-dimensional, M-mode, and color Doppler imaging modalities.

### Digital stethoscope specifications

A WP-100 digital stethoscope was used for auscultation and heart sound sample collection. This device integrates Bluetooth BLE 5.0, enabling efficient and seamless wireless data acquisition. It utilizes an MEMS microphone as the primary sensor for capturing acoustic signals. The stethoscope operates in two specialized modes optimized for distinct frequency ranges: “Heart Mode” (50–300 Hz) for cardiac sounds and “Lung Mode” (100–1200 Hz) for respiratory sounds. Audio playback was configured at 16 kHz, mono, in a 16-bit PCM format, allowing direct auscultation through headphones connected to the stethoscope’s integrated USB-C connector and facilitating sound reproduction for clinical assessment.

A companion application was used for the audio recordings and AI analysis. The device downsamples audio to 8 kHz, mono, 16-bit PCM format to ensure stable BLE connectivity and optimize the data for subsequent filtering. Upon transmission to the application, advanced filtering techniques were applied to minimize noise and friction artifacts and enhance the audio quality for AI-based murmur detection analysis.

### Deep learning algorithm development

#### Data acquisition and allocation

A total of 2,269 recordings over 20 h were collected from 406 patients. Each heart-sound recording was annotated based on an assessment conducted by a trained veterinarian using a standard stethoscope. The dataset was divided into training, validation, and test sets in a 7:2:1 ratio, with 1,972 recordings allocated to the training set, 548 recordings from 69 dogs to the validation set, and 297 recordings from 60 dogs to the test set. To ensure balanced representation, the class distribution within each set was allocated to preserve the approximate 3:7 ratio of ‘non-murmur’ to ‘murmur’ cases inherent in the total collected dataset, with distributions of 25.2% and 74.8% in the training set, 24.5% and 75.5% in the validation set, and 36.7% and 63.3% in the test set. The test set was held exclusively for the final performance evaluation, whereas the validation set was used during training to monitor and optimize the model classification performance.

#### Preprocessing

**Fig. 2:**
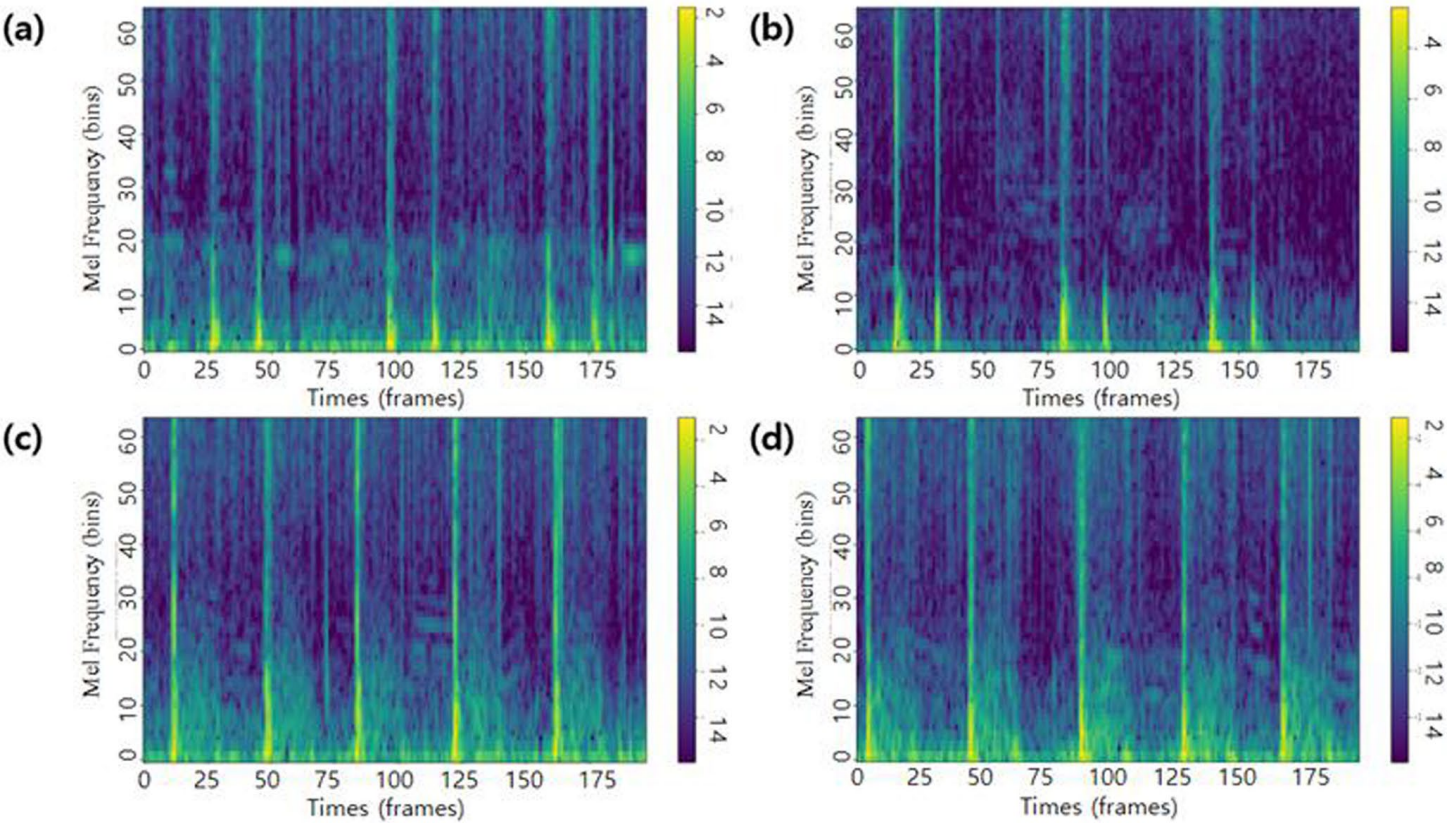
Examples of Fbank. The spectrocardiograms demonstrate **(a**,** b)** normal heart sound and **(c**,** d)** murmur. Each spectrogram visualizes approximately 2 s of audio, with time represented on the horizontal axis (~ 200 frames) and 64 Mel frequency bins on the vertical axis. Color intensity reflects spectral energy in decibels (dB), with brighter regions indicating higher energy levels associated with heart sound components

Each audio recording was divided into fixed-length segments of 8 s to ensure uniform input data. A standard normalization technique was applied to the audio signals to enhance consistency. This normalization, applied per recording and performed automatically by a function in Torchaudio, scales the sample values of each file to the range of −1.0 to 1.0. Each segment was transformed into a feature representation using either a filter bank (Fig. 2) or the mel-spectrogram method, determined by the specified parameters. The transformation settings were standardized with a sample rate of 8,000 Hz, FFT size of 1,024 and a window length of 1,024. Variable parameters during training included the hop length, set to either 80 or 256; the number of mel bands, set to 64 or 80; and an optional filtering step, which, when set to True, applied a biquad filter with a frequency range of 10–500 Hz. Data augmentation was performed using SpecAugment (Park et al. 2019) when specified; otherwise no augmentation was used if not configured. These preprocessing steps were designed to capture the key frequency characteristics of heart sounds while minimizing noise and irrelevant variations. Various parameter combinations were tested on heart sounds to determine the most suitable configuration for the model performance.

#### Model development

The CNN6 model was designed to classify heart-sound recordings into ‘non-murmur’ and ‘murmur’ categories. This architecture was chosen after comparative evaluation against deeper models, emphasizing its role as the most suitable model balancing accuracy and efficiency. The model’s inherent simplicity was prioritized because it minimizes the risk of overfitting and allows for efficient optimization of feature extraction for the binary classification task. Its architecture involves multiple convolutional layers, each using 5 × 5 kernels, followed by batch normalization and ReLU activation functions. The filter sizes of the convolutional layers were progressively increased to 64, 128, 256, and 512, enhancing the model’s ability to extract relevant features. Max-pooling layers with 2 × 2 kernels were implemented after each convolutional block to decrease the spatial dimensions and mitigate overfitting. The feature maps were then aggregated using a global pooling layer and passed into a fully connected classifier.

The model was initialized using random weights and optimized using the Adam optimizer. Training was performed with an initial learning rate of 0.001 and a batch size of eight over 200 epochs. Early stopping based on the validation loss was used to avoid overfitting, with the mechanism configured in ‘min’ mode and set to a patience value of 10 epochs. The cross-entropy loss function was employed as the objective in all the training experiments.

The complete model outputs a classification of either ‘non-murmur’ or ‘murmur’. Final evaluations of the test set provided an extensive assessment of the ability of the model to distinguish between these two classes.

#### Performance evaluation

The performance of the deep learning model was assessed using three metrics–accuracy, sensitivity, and specificity–from which the positive and negative likelihood ratios were derived. To measure these metrics, the numbers of true positives (TP), true negatives (TN), false positives (FP), and false negatives (FN) were calculated for each class.

$$\:Accuracy=\frac{TP+TN}{TP+FP+FN+TN}$$

$$\:Sensitivity=\:\frac{TP}{TP+FN}$$

$$\:Specificity=\:\frac{TN}{TN+FP}$$

### Deep learning model evaluation and veterinarian comparison test

To assess the performance of the trained algorithm, veterinarians annotated the recordings, allowing a direct comparison with AI. Among the algorithm models, the seed model that demonstrated the best performance was selected for comparison. Echocardiography was conducted in dogs with heart murmurs detected at the mitral valve using a standard stethoscope to confirm mitral regurgitation and culminate in the diagnosis of MMVD.

The heart sounds collected for the test set were subjected to a manual quality evaluation, and the results were assigned to one of three tiers: good, fair, and poor. Patients with poor-quality recordings were excluded. Quality was determined based on auditory assessment: *good* recordings had clearly audible heart sounds without noticeable noise; *fair* recordings contained mild noises but heart sounds remained distinguishable; and *poor* recordings had either excessive noise or poor signal quality that made the heart sounds indistinguishable. Up to six samples were included for each subject to evaluate the AI performance.

The expert annotators listened to all de-identified recordings using high-quality headphones in a quiet environment and were blinded to both the algorithmic results and the ground truth. All annotators were also blinded to the patients’ clinical information, including age, breed, and echocardiographic findings. The expert annotators were veterinarians with backgrounds in internal medicine who were proficient in auscultation using a standard stethoscope. Prior to annotation, veterinarians participated in a training session using ground-truth-labeled heart sounds to become acquainted with the auditory characteristics of the recordings. They then annotated the presence or absence of murmurs in the veterinarian comparison set, with the option of listening to the recordings as many times as necessary before making a judgment.

Although formal inter-observer agreement statistics were not calculated, all five veterinarians followed the same standardized annotation protocol under identical conditions and received unified training. This consistent methodology helped ensure reliability in labeling and minimized subjectivity in murmur identification.

### Statistical analysis

Statistical analysis and data visualization were conducted in Python 3.8 utilizing standard libraries, including Numpy, Pandas, Seaborn, Matplotlib, Scikit-learn, and Torchaudio. The model evaluation metrics, including accuracy, sensitivity, and specificity, were calculated using TorchMetrics, whereas the mean and standard deviations were computed using Numpy. The 95% confidence intervals (CIs) were calculated using SciPy. Likelihood ratios were computed using custom calculations implemented in Torch. In the veterinarian comparison test, McNemar’s test was performed using the R-4.4.1 software to compare the sensitivity and specificity between each veterinarian and the model. All the experimental environments are listed in Table 2.

**Table 2:** Experimental environments

OS	Ubuntu 22.04.2 LTS
CPU	13th Gen Intel(R) Core(TM) i9-13900KF
RAM	62 GB
GPU	$2\times NVIDIA\;Geforce\;RTX\;4090$
CUDA Version	CUDA 11.8

## Results

### Participants characteristics

The demographic and clinical characteristics of the study participants are summarized in Table 3. The subjects had a mean age of 10.62 ± 3.51 years. Breeds included in the other categories included Pug, Shetland sheepdog, Bulldog, Shar-pei, Golden retriever, Standard poodle, Pompitz, Italian greyhound, Bedlington terrier, White terrier, Doberman pinscher, Goldendoodle and Jindo.

**Table 3:** Characteristics of study subjects. Data for overall age is presented as mean ± SD. Numbers are presented for specific categories of sex, neuter state, breed, age and cardiovascular diseases. Left-sided heart sounds are auscultated over the mitral, aortic, and pulmonic valve regions on the left side of the thorax, whereas right-sided heart sounds are detected at the tricuspid valve region on the right side of the thorax. The listed diseases were diagnosed based on findings from echocardiography. Right-sided heart sound information was not obtained from a total of 109 dogs. MMVD; myxomatous mitral valve disease, ACVIM; American College of Veterinary Internal Medicine, PH; pulmonary hypertension, TVI; tricuspid valve insufficiency, PDA; patent ductus arteriosus, VSD; ventricular septal defect

Characteristics	All subjects	Left-sided heart sound		Right-sided heart sound	
		Normal	Murmur	Normal	Murmur
Total subjects	406	179	227	132	165
**Sex**					
Male	220	95	125	64	94
Female	186	84	102	68	71
**Neuter State**					
Intact	20	12	8	8	5
Neutered	386	17	219	124	160
**Breed**					
Maltese	121	43	78	31	56
Poodle	55	26	29	20	22
Pomeranian	49	23	26	17	16
Shih-tzu	40	12	28	8	23
Chihuahua	20	7	13	5	12
Yorkshire Terrier	15	10	5	5	3
Bichon Frise	11	9	2	6	2
Cocker Spaniel	7	1	6	1	4
Spitz	7	2	5	0	4
Miniature Pinscher	6	4	2	3	1
Schnauzer	6	2	4	2	4
Dachshund	5	0	5	2	3
Welsh Corgis	4	4	0	1	0
Labrador Retriever	4	3	1	2	1
Maltipoo	3	3	0	3	0
Scottish Terrier	2	2	0	0	0
Boston Terrier	2	1	1	1	0
French Bulldog	2	2	0	1	0
Coton de Tulear	2	0	2	0	2
Pekingese	2	0	2	0	2
Mixed	29	13	16	14	9
Others	14	12	2	10	1
**Age**					
< 7 y	72	54	18	34	13
7–9 y	75	44	31	40	18
9–11 y	82	33	49	21	35
11–13 y	89	28	61	20	49
13–15 y	68	14	54	15	38
> 15 y	20	6	14	2	12
Mean ± SD, y	10.62 ± 3.51	9.09 ± 3.63	11.82 ± 2.91	9.28 ± 3.41	11.96 ± 2.90
**Diseases**					
MMVD	214	5	209	30	153
ACVIM stage B1	87	4	83	22	55
ACVIM stage B2	81	0	81	6	65
ACVIM stage C	42	1	41	2	30
ACVIM stage D	4	0	4	0	3
PH	8	2	6	2	5
TVI	1	0	1	0	1
Cardiac mass	3	2	1	2	1
PDA	3	0	3	0	3
VSD	2	0	2	0	2

### Deep learning development result

The CNN6 model was developed to classify heart-sound recordings into ‘non-murmur’ and ‘murmur’ categories. Sensitivity and specificity were both prioritized in the model selection process because of their critical roles in clinical diagnostic sensitivity, ensuring accurate detection of murmur cases and specificity in reducing false positives. The best-performing model was selected based on the highest average sensitivity and specificity scores in the validation set, ensuring a balanced approach that prioritized both metrics.

During training and validation, the model demonstrated progressive improvements across all key performance metrics. The CNN6 model was evaluated across four random seeds. The reported metrics reflected the average performance to ensure robustness. The model achieved an accuracy of 99.6% ± 0.03% on the training set and 87.72% ± 0.74% on the validation set, indicating effective model generalization.Sensitivity reached 99.63% ± 0.02% on the training set and 90.1% ± 1.49% on the validation set. The specificity was 99.52% ± 0.15% on the training set and 78.05% ± 3.7% on the validation set, indicating its capability to correctly identify negative cases.

**Fig. 3:**
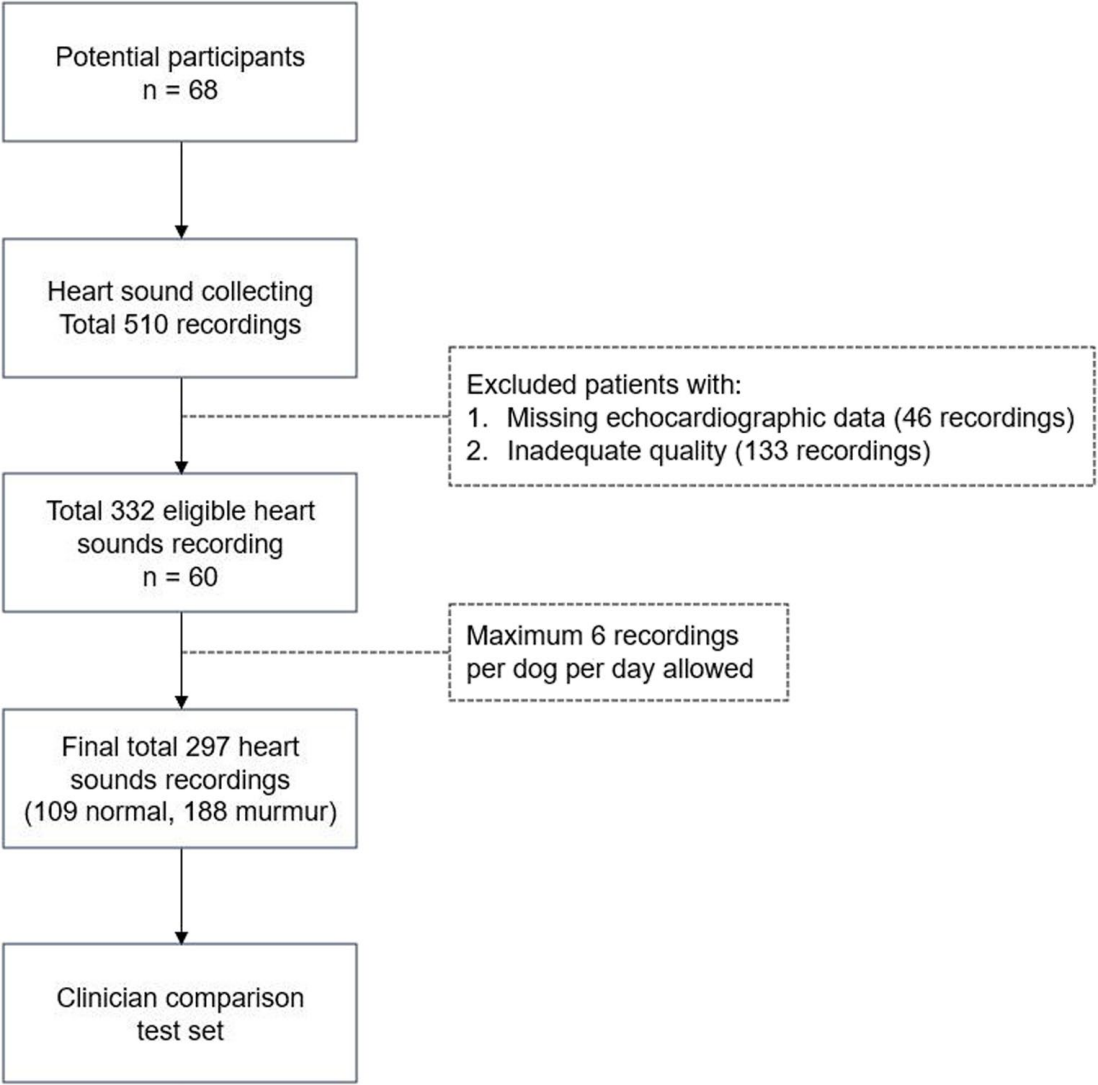
Flowchart illustrating the selection process for the test set used to compare the deep learning model and clinician performance. A total of 510 recordings were collected from 68 dogs, of which 179 were excluded, resulting in 332 eligible recordings. After limiting the dataset to six recordings per dog per day, the final dataset comprised 297 recordings, consisting of 109 non-murmur and 188 murmur recordings

For the test set, 510 recordings were collected at the mitral valve location from 68 dogs (Fig. 3). The heart sound quality was categorized into three tiers: 170 recordings (33%; 52 normal heart sounds and 118 murmurs) were classified as good quality; 207 recordings (41%; 70 normal heart sounds and 137 murmurs) as fair quality; and the remaining 133 recordings (26%; 58 normal heart sounds and 75 murmurs) as poor quality, which were excluded from the test set. Of the remaining 377 recordings, 46 recordings with missing echocardiographic data were excluded. For each subject, up to six heart-sound recordings from the same date were used, prioritizing those classified as good quality.

In total, 297 recordings (109 normal heart sounds and 188 murmurs) from 60 dogs were assigned to the test set. The algorithm classified the recordings into “no murmur” and “murmur,” and the results were directly compared to the ground truth labels. The model achieved an accuracy of 87.84% ± 0.66% and a sensitivity of 86.37% ± 2.14%, validating its robustness and reliability in distinguishing between murmur and non-murmur recordings. Specificity was recorded at 90.37% ± 3.16%, underscoring the model’s ability to correctly identify non-murmur cases. The best-performing model was selected based on the highest accuracy and sensitivity, with sensitivity prioritized to reduce the risk of false negatives, which is essential for patient safety in clinical settings.

### Veterinarian comparison test

**Fig. 4:**
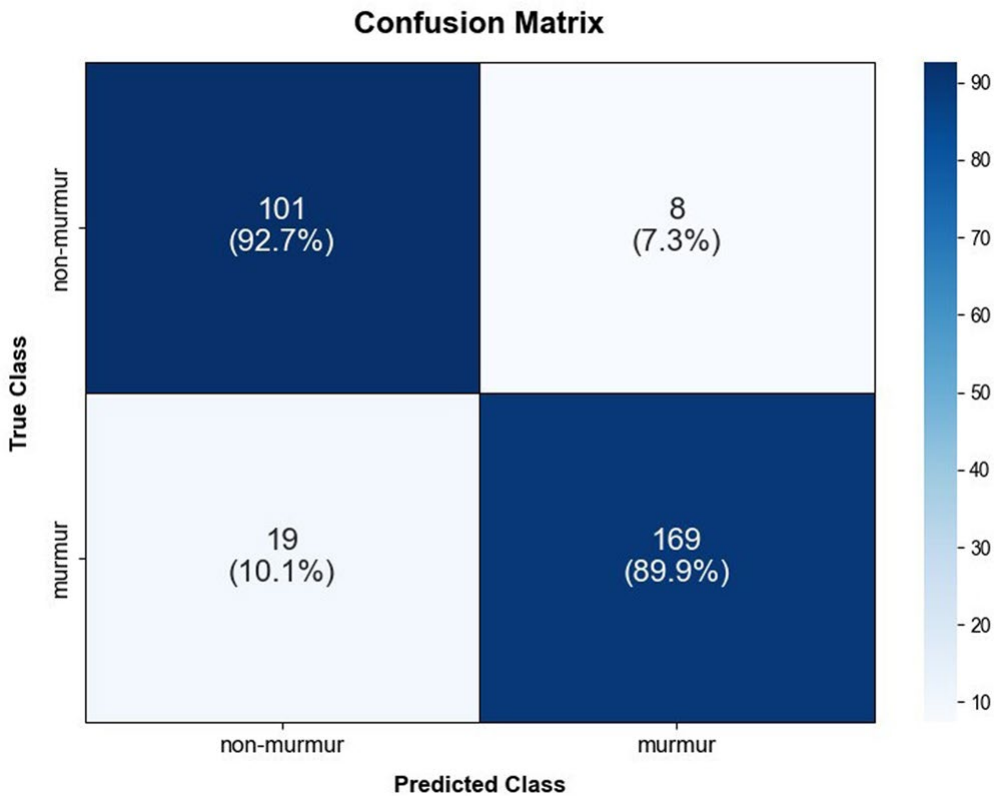
Performance evaluation of deep learning model on a test set. The confusion matrix depicts the percentage of predictions across each category in the test set. The “true class” represents the ground truth, verified by standard stethoscope, while the “predicted class” reflects the output generated by the deep learning model, indicating the model’s capability to replicate the ground truth. Among 297 test recordings, the model correctly classified 169 murmur and 101 non-murmur samples, achieving a sensitivity of 89.9%, a specificity of 92.7%, and an overall accuracy of 90.9%. The high diagonal dominance of the matrix demonstrates the model’s robust capability to replicate ground truth and its potential utility for objective murmur screening in clinical practice

The performance of the model used in the comparison test was evaluated, with a sensitivity of 89.9%, a specificity of 92.7%, and an accuracy of 90.9% (Table 4). The classification results are summarized in the confusion matrix shown in Fig. 4. The performances of the five veterinarians were evaluated, with an average sensitivity of 86.9%, a specificity of 84.6%, and an accuracy of 86.1% (Table 4).

**Table 4:** Performance of independent clinical annotators compared to the algorithm. The table presents the sensitivity, specificity, and accuracy for each clinician and the model, along with their 95% confidence intervals, as well as the average sensitivity, specificity, and accuracy across all clinicians. Confidence intervals are shown in brackets as [lower–upper]. All evaluations were based on the test dataset (*n* = 297). CI; Confidence Interval. *p* < 0.05(*), *p* < 0.01(**) represent statistical significance compared with model values

Annotators	Evaluation matrix				
	Sensitivity [95% CI], %	*p*	Specificity [95% CI], %	*p*	Accuracy [95% CI], %
Annotator A	93.6 [89.1–96.7]	0.105	73.4** [64.1–81.4]	< 0.001	86.2 [81.7–89.9]
Annotator B	78.2** [71.6–83.9]	< 0.001	78.0** [69.0–85.4]	0.001	78.1 [73.0–82.7]
Annotator C	86.2 [80.4–90.8]	0.072	93.6 [87.2–97.4]	0.500	88.9 [84.8–92.2]
Annotator D	84.0* [78.0–89.0]	0.010	87.2 [79.4–92.8]	0.132	85.2 [80.6–89.0]
Annotator E	92.6 [87.8–95.9]	0.166	90.8 [83.8–95.5]	0.402	91.9 [88.2–94.8]
**Average %**	86.9	-	84.6	-	86.1
**Algorithm** **(95% CI)**,** %**	89.9 [84.7–93.8]	-	92.7 [86.0–96.8]	-	90.9 [87.0–93.9]

**Fig. 5:**
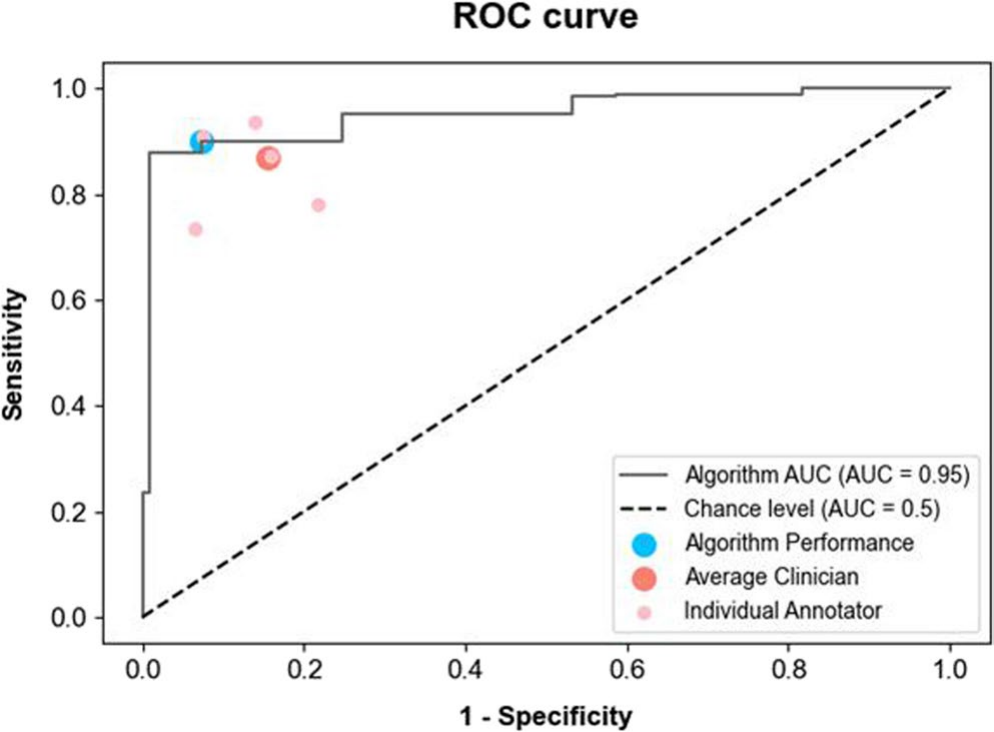
Comparison of performance between the model, the individual clinicians and the average performance of the clinicians at detecting murmurs. The model receiver operating characteristics (ROC) curve is shown with its performance (blue marker). The ROC curve of the model is generated by varying the discrimination threshold. The performances of individual veterinarians and their average are indicated by markers (light pink = individual, red = average). The proximity of the clinical data points to the model’s ROC curve demonstrates that the algorithm achieved a diagnostic performance comparable to the veterinarians in detecting heart murmur. AUC; area under the curve

The diagnostic performance of the model was demonstrated using the receiver operating characteristic (ROC) curve shown in Fig. 5. The individual performance of each veterinarian, along with the average veterinarian performance, are represented by dots on the graph. The murmur detection algorithm (Fig. 5; area under the curve = 0.95) exhibited a performance comparable to that of veterinarians (represented by light pink in Fig. 5). The veterinarians’ performance points are located near the ROC curve of the model, indicating that the algorithm achieved similar levels of sensitivity and specificity.

Using McNemar’s tests, performance differences between the model and individual veterinarians were assessed (Table 4). Annotators B (*p* < 0.001) and D (*p* = 0.010) showed significantly lower sensitivity than the model, while Annotators A (*p* < 0.001) and B (*p* = 0.001) showed significantly lower specificity. Overall, the model’s diagnostic performance was comparable to the range observed among clinicians and demonstrated high concordance with auscultation-based labels. To assess the reliability of the veterinarian annotations used for this comparison, inter-observer agreement among the five annotators was calculated using Fleiss’ κ, yielding a value of 0.614. This indicates substantial agreement (Landis and Koch 1977), supporting the consistency and reliability of the labeling process.

In conclusion, the model achieved clinician-level performance in differentiating murmurs from normal heart sounds, primarily representing alignment with established auscultation patterns.

## Discussion

Recent advancements in AI, particularly deep learning, have revolutionized various aspects of medical diagnostics, including cardiovascular disease detection. In the context of cardiovascular disease diagnosis, deep learning plays a critical role in analyzing complex physiological signals such as electrocardiograms (Hannun et al. 2019; Mathews et al. 2018; Smith et al. 2019), cardiovascular imaging (Abdar et al. 2019; Slomka et al. 2020; Wang et al. 2019), and heart sounds (Arjoune et al. 2023; Chorba et al. 2021; DeGroff et al. 2001; Elola et al. 2023; Khan et al. 2023; Nguyen et al. 2023; Zhao et al. 2023). These advancements demonstrate the ability of AI to efficiently process large amounts of unstructured data and detect subtle patterns associated with heart conditions. In human medicine, deep learning algorithms have been successfully employed for heart sound analysis, achieving a diagnostic accuracy comparable to that of cardiologists —for example, up to 92.6% classification accuracy (Ahmad et al. 2019), 90.0% sensitivity when excluding grade 1 murmurs (Chorba et al. 2021), and 84.7% accuracy compared to an average clinician accuracy of 77.9% (Prince et al. 2023). However, despite these successes in human healthcare, the application of deep learning in veterinary medicine remains limited, particularly in cardiac auscultation. This study addresses this gap by developing and evaluating a deep-learning based heart murmur detection model tailored for canine patients.

This study highlights the potential of the developed model as a diagnostic screening tool for MMVD. Given the reported prevalence of MMVD in middle aged dogs (40%) (Borgarelli and Buchanan 2012; Whitney 1974), the model’s negative likelihood ratio (12.25) reduces the post-test probability to 6.8%, wheareas the positive likelihood ratio (0.11) increases it to 89.1% (Deeks and Altman 2004; McGee 2002).Therefore, while a negative result warrants further diagnostic testing owing to the remaining possibility of the disease, a positive result strongly suggests the presence of MMVD. As a screening tool, this model offers a cost-effective and rapid method for early filtering, particularly in high-risk patients or large-scale screening scenarios, enhancing clinical efficiency in veterinary cardiology.

To validate our architectural choice, comparative experiments were performed against the deeper ResNet-38 and the transformer-based AST models. These comparison models were tested using the same fixed hyperparameters as CNN6. The comparison confirmed that the CNN6 is the most suitable architecture, achieving the highest diagnostic accuracy while requiring the lowest computational resources. The more complex ResNet-38 and AST models achieved significantly lower diagnostic accuracy (Table 5) compared to CNN6, confirming that increased model depth did not translate to improved generalization on our specific veterinary dataset. Furthermore, the efficiency metrics solidified the choice: CNN6 utilizes 4.3 million trainable parameters and operates with an average inference time of 0.505 ms, which is over four times faster than the alternatives. This validated trade-off between superior accuracy and extreme computational efficiency is essential for the practical implementation of the system in veterinary practice.

**Table 5:** Comparative performance and computational efficiency of deep learning architectures. Performance comparison of the CNN6 model against deeper architectures tested on the independent canine heart sound test set. The CNN6 model achieved the highest diagnostic performance while maintaining the lowest computational cost

Architectures	Test Accuracy (%)	Test Sensitivity (%)	Test Specificity (%)	Trainable Parameters (M)	Inference Time (ms)
CNN6	87.84	86.37	90.37	4.3	0.505
ResNet-38	46.83	37.83	62.5	72.7	2.173
AST	56.21	61.44	47.11	85.2	2.633

Despite these promising results, several limitations warrant discussion. First, while the model demonstrated strong performance in detecting heart murmurs, its diagnostic sensitivity and specificity for MMVD screening may be influenced by the reliance on auscultation findings as the ground truth. Because echocardiography was only performed in dogs with audible murmurs, subclinical cases without detectable murmurs may have been classified as non-murmur, potentially leading to an overestimation of specificity. Nevertheless, this approach aligns with the study’s primary objective—to develop an AI model that replicates clinical auscultation patterns rather than providing a definitive echocardiographic diagnosis. Future studies should integrate both auscultation and echocardiographic confirmation to improve early detection. Additionally, because standard stethoscopic findings were used as the ground truth, examiner subjectivity may have influenced the results. Moreover, as both labeling and evaluation were based on auscultation findings, the evaluation framework is susceptible to circular validation bias, since the model was assessed against the same diagnostic modality used for labeling. This limitation implies that the reported metrics primarily reflect agreement with clinical auscultation rather than absolute diagnostic accuracy. To mitigate these issues, future research should record auscultation data and establish the ground truth through consensus or voting among multiple veterinarians and incorporate echocardiographic validation to reduce inter-observer variability and enhance diagnostic reliability.

Another potential limitation is the inclusion of multiple heart sound recordings from the same individuals, which may introduce a risk of overfitting (He and Garcia 2009; Japkowicz 2000). Although efforts were made to mitigate this risk, such as ensuring that each subject was included in only one dataset (Bengio et al. 2017; Domingos 2012) and employing SpecAugment to diversify the data (Park et al. 2019), the model demonstrated superior performance when SpecAugment was excluded. SpecAugment, a technique that applies time and frequency masking to the input feature maps, was utilized to test for robustness against transient noise and spectral shifts. Comparative analysis showed a significant drop in specificity from 78.05% to 58.82, which offsets the minor gain in sensitivity of 2.99% and reduced the overall accuracy by 1.41%. This large decrease suggests that the augmentation strategy obscured the critical, low-frequency signatures and harmonics essential for distinguishing a non-murmur heart sound from a murmur. Murmurs in PCG data are concentrated in the low frequency range and the masking process, designed for high-redundancy speech, likely removed the subtle yet vital acoustic markers, making non-murmur segments acoustically ambiguous. This suggests that the original dataset contained sufficient variability and that certain augmentation techniques may have introduced unnecessary distortions. Future studies should explore alternative augmentation strategies and validate model performance across larger, independent datasets to enhance generalizability.

In addition, about 26% of recordings in the comparison test set were excluded due to poor signal quality—such as severe background noise or motion artifacts—that made the heart sounds difficult to interpret even by human experts. Although this exclusion was necessary to ensure consistent signal interpretation, it may limit the model’s applicability under real-world conditions where recording quality varies.

Finally, the dataset predominantly consisted of small-breed dogs, reflecting the demographic characteristics of the companion animal population in Korea. While this composition represents real clinical cases, it may restrict the generalizability of the model to populations with different breed compositions or lifespan patterns. Future studies that include a more representative proportion of medium- and large-breed dogs could further validate the model’s applicability across diverse canine populations.

This study demonstrates the potential of a deep learning model integrated with a digital wireless stethoscope for detecting heart murmurs, highlighting its role in the early diagnosis of heart diseases such as MMVD. While the primary focus was murmur detection, future advancements could incorporate disease severity and staging to provide a more comprehensive assessment, aiding in personalized treatment planning. For example, detecting a shift toward more advanced disease stages could prompt clinicians to shorten follow-up intervals, initiate additional diagnostic testing, or consider the early introduction or adjustment of pharmacologic therapy based on individualized risk profiles.

MMVD can progress to pulmonary edema, causing severe respiratory distress. In such cases, echocardiography may exacerbate patient stress, while biomarkers like serum N-terminal pro-B-type natriuretic peptide offer only indirect insights (Oyama et al. 2009). A well-developed AI model could enable immediate, non-invasive assessment, improving patient safety, reducing costs, and optimizing medical resource use. Additionally, real-time heart sound monitoring in home settings could provide early alerts for hospital visits before clinical symptoms emerge. As more data accumulates, this model could contribute significantly to heart disease research and drug development.

Collectively, our findings demonstrate that the deep learning model, utilizing PCG data recorded via a digital wireless stethoscope, achieves diagnostic accuracy comparable to that of experienced veterinarians. By effectively identifying subtle murmur patterns, the model provides a reliable and objective approach to heart murmur detection, minimizing inter-observer variability inherent in traditional auscultation. Its ability to deliver consistent and reproducible diagnoses has significant clinical implications, particularly in settings with limited access to veterinary specialists. Additionally, the integration of this AI model into a digital stethoscope enhances its applicability in telemedicine, supporting remote cardiac evaluations and early disease screening. In daily clinical practice, such a system could assist veterinarians by providing real-time murmur screening during routine examinations or by enabling remote consultations through tele-auscultation. However, successful implementation will require consideration of practical factors such as device cost, data management, and compliance with data protection regulations to ensure secure and sustainable clinical adoption. The ability to conduct assessments in a low-stress environment, such as a dog’s home, may further improve diagnostic accuracy by reducing patient anxiety and its potential impact on cardiac function. This advancement broadens the scope of cardiovascular screening in dogs, facilitating earlier detection and intervention.

In conclusion, this study underscores the potential of deep learning-driven auscultation as a valuable tool for early heart disease detection in dogs, particularly for MMVD screening. By leveraging a digital wireless stethoscope, the model provides an objective, reproducible, and accessible method for heart murmur detection, addressing key limitations of traditional auscultation. While further validation through larger, independent datasets and integration with echocardiographic confirmation is needed, this AI-assisted approach holds promise for enhancing diagnostic accuracy, facilitating telemedicine applications, and improving patient outcomes. As advancements in deep learning and veterinary cardiology continue to evolve, AI-driven auscultation could play a pivotal role in the future of veterinary medicine, enabling earlier intervention and personalized care for canine cardiac patients.

## Data Availability

No datasets were generated or analysed during the current study.
